# Investigating the Implementation and Impact of AI-Assisted Fall Prevention in Hospitals: Protocol for a Multicenter, Multimethod Observational Study in Sweden (SAFE)

**DOI:** 10.2196/84294

**Published:** 2026-05-04

**Authors:** Elin Siira, Anna Gyberg, Ingrid Larsson, Marcus Rosenburg, Petra Svedberg, Kerstin Ulin, Helle Wijk, Thomas Brezicka, Malin Hansson, Jenny Hallgren, Jens Nygren

**Affiliations:** 1 School of Health and Welfare, Halmstad University, Box 823, Halmstad, 301 18, Sweden, 46 706924613; 2 Department of Medicine, Geriatrics and Emergency Medicine, Sahlgrenska University Hospital/Östra, Gothenburg, Sweden; 3 Institute of Health and Care Sciences, Sahlgrenska Academy, University of Gothenburg, Gothenburg, Sweden; 4 Department of Architecture and Civil Engineering, Chalmers University of Technology, Gothenburg, Sweden; 5 Department of Quality Assurance, Sahlgrenska University Hospital, Gothenburg, Sweden; 6 Region Västra Götaland, Research, Education, Development and Innovation, Primary Health Care, Gothenburg, Sweden; 7 School of Health Sciences, University of Skövde, Skövde, Sweden

**Keywords:** artificial intelligence, fall, prevention, health care leaders, health care professionals, hospital, implementation, impact, patient safety, patients, resource use, work, work environment

## Abstract

**Background:**

Artificial intelligence (AI) has the potential to enhance patient safety, particularly in the prevention of in-hospital falls. Recent advances in sensor-based AI systems allow for the analysis of complex, multimodal data to generate real-time alerts, enabling health care professionals to intervene before a fall occurs. By shifting from reactive responses to proactive risk management, these technologies may enable reductions in fall incidence and improvements in care outcomes. As a result, hospitals across Europe are increasingly adopting such systems. Nevertheless, empirical evidence concerning their routine implementation remains limited, particularly concerning their impact on patient safety, clinical workflows, and the usage of health care resources. Addressing these gaps is essential for effective and sustainable integration into hospital care.

**Objective:**

This paper outlines the protocol for the multicenter, multimethod project SAFE (Safe AI-Assisted Fall Prevention Through Evidence), which investigates the implementation and impact of AI-assisted fall prevention in Swedish hospitals.

**Methods:**

The research project is a collaboration between Halmstad University and hospitals in the Västra Götaland Region (VGR) and will, during 2026‐2028, investigate an ongoing large-scale AI system implementation in VGR hospitals, covering up to 2400 patient beds. Using surveys, interviews, observations, and a retrospective study, it will track the implementation and impact over time. Two learning laboratories involving patients, their relatives, and health care professionals will be conducted to codevelop strategies for the implementation of AI-assisted fall prevention.

**Results:**

The project will provide evidence-based insights and practical guidance on AI-assisted fall prevention. The findings will be relevant not only to patients, health care professionals, and hospital organizations, but also to policymakers and stakeholders involved in the digital transformation of health care.

**Conclusions:**

Although VGR serves as the primary research setting, the project’s results will inform future similar initiatives in Sweden and offer transferable lessons for other health care systems internationally. This study will contribute to the evidence base on AI-assisted fall prevention in health care, supporting the responsible and scalable integration of such systems across diverse health care environments.

## Introduction

### Background

Fall-related injuries rank among the most frequent and costly adverse events in hospital settings, placing a significant burden on health care systems worldwide [1,2]. These events usually have multiple causes, arising from the interaction of several patient-related factors such as advanced age, low body weight or undernutrition, and psychiatric conditions [3-5], as well as clinical conditions including dementia and delirium, which further heighten the risk. Falls are particularly prevalent in neurological and geriatric wards, with the majority occurring in patient rooms, most often during ambulation near beds and chairs [6].

In addition to physical harm, falls can lead to considerable psychological distress, including anxiety, depression, loss of independence, and diminished quality of life. In severe cases, they are also associated with prolonged hospitalization and increased mortality [1]. The economic implications are substantial [7,8], encompassing not only the immediate costs of acute treatment and rehabilitation, but also expenses for municipal and long-term care, home modifications, and significant informal caregiving. Broader societal impact includes decreased productivity for patients and caregivers, diminished health-related quality of life, and premature mortality in the most severe cases.

Evidence suggests that 20%‐30% of inpatient falls are preventable through the implementation of targeted interventions [9]. However, the actual effectiveness of such interventions depends on factors including contextual suitability, fidelity of implementation, and the allocation of resources relative to other health care priorities. Existing prevention strategies encompass patient-initiated measures (eg, call bells and alarms to alert staff), health care professional-led interventions (eg, fall risk assessments, intentional rounding, and the use of sitters), and technology-based approaches (eg, alarms, nonslip socks, and bed rails). While multicomponent interventions generally demonstrate superior effectiveness, evidence from hospital settings remains inconclusive, particularly regarding long-term outcomes [2,10]. This persistent gap highlights the necessity for further research to design, implement, and evaluate innovative, evidence-based fall prevention strategies, including those incorporating artificial intelligence (AI).

### AI-Assisted Fall Prevention

Recent advancements in AI have enabled the analysis of large, multimodal sensor datasets to identify early indicators of falls and generate real-time alerts for health care professionals [11,12]. Such AI-driven systems have the potential to reduce the incidence of falls by allowing staff to prioritize their actions and respond rapidly when a high-risk situation emerges. This, in turn, supports a shift toward more proactively managing fall risks [11,13]. Moreover, some AI models can be continuously updated as new data accumulate, allowing for iterative improvements in predictive accuracy and overall system performance [12,13].

While these innovations demonstrate considerable safety potential, they remain in the early stages of development, and evidence regarding their implementation and routine use is currently limited [12,13]. In contrast to conventional technologies, AI systems not only structure but can also identify patterns in data, thereby influencing clinical knowledge production and decision-making processes. As such, their implementation represents a complex sociotechnical challenge, necessitating adaptations to clinical workflows, data governance structures, and mechanisms for knowledge sharing within health care organizations [14-18]. Careful consideration of these factors is essential to support effective integration and to realize improvements in patient outcomes.

Different AI-assisted fall-prevention interventions have been piloted internationally, demonstrating promising outcomes. In Sweden, an innovation project conducted within routine clinical practice reported a reduction in falls of up to 67% [19]. Health care staff noted improved early detection of fall risk and a reduced need for continuous in-room observation. Additional reported benefits included faster response times to alarms, enhanced monitoring during nighttime hours, and potential cost savings attributed to decreased supervision requirements, fewer additional care days, and a reduction in diagnostic procedures such as x-rays [19]. Similarly, pilot studies in Switzerland highlighted several advantages, including reduced nursing workload and effective integration into routine clinical workflows [20-22].

Concurrently, participants in both settings identified several limitations associated with the interventions, including false alarms, alarm fatigue, elevated noise levels, and various technical challenges. In the Swedish context, difficulties were noted in accurately linking alarms to the correct patient, with the AI system occasionally failing to differentiate between patients, visitors, and staff members [19]. In the Swiss context, some staff members reported a sense of being under surveillance [21], and approximately 30% of falls were not detected due to atypical movement patterns, such as rolling from low beds [20].

Overall, while these pilot studies suggest potential benefits, robust evidence remains limited regarding the broader impacts of these systems. This includes their alignment with existing work practices, their effects on the work environment, and their influence on patients’ care experiences, clinical outcomes (including fall incidence), and resource usage. The implementation of AI systems also raises questions about human oversight and professional autonomy, as staff retain the ability to exercise clinical judgment and may adapt their interaction with the system, such as setting devices aside or silencing alerts to maintain a sustainable work environment [23]. In addition, concerns related to the fairness of AI systems remain insufficiently explored, particularly regarding whether AI-assisted fall prevention varies across patient subgroups. Moreover, the organizational conditions necessary for successful hospital-wide implementation have yet to be clearly defined. Considering all these dimensions of AI-assisted fall prevention, from clinical outcomes and resource use to the everyday experiences and well-being of health care professionals and patients, is essential for supporting implementations that enhance patient safety, ensure good working conditions, and promote the appropriate use of resources.

Evidence on the implications, effects, and real-world implementation of AI-assisted fall prevention in hospital settings remains scarce. Building on insights from the pilot studies conducted in Sweden and Switzerland [20-22], our research aims to generate evidence-based knowledge through a comprehensive evaluation embedded within an ongoing region-wide implementation of an AI-assisted fall-prevention intervention in the Västra Götaland Region (VGR), Sweden. VGR is one of Europe’s largest health care organizations, both in terms of population served—approximately 1.8 million residents—and the scale of its infrastructure and workforce comprising more than 50,000 employees in the health care sector. The current implementation comprises all hospitals in VGR and will engage a substantial number of patients, health care professionals, and clinical wards. Despite the scale of this initiative, given the emerging nature of AI-assisted fall prevention, there remains a paucity of robust evidence regarding its effects and its broader implications for care delivery, workforce conditions, and resource usage, underscoring the need for further empirical investigation.

### Aim of the Project

The SAFE (Safe AI-Assisted Fall Prevention Through Evidence) project aims to generate evidence-based insights into the implementation and impact of AI-assisted fall-prevention within hospital settings. It addresses a critical knowledge gap by advancing understanding of how such AI systems function in routine care, how they are adopted and normalized, and how they influence patient and organizational outcomes. In addition, this project will, through collaboration with health care professionals and patients, develop practice-oriented strategies to support hospitals in implementing AI for fall prevention in ways that promote patient safety, safeguard sustainable working conditions, and enable appropriate use of resources.

Specific research questions (RQ) are as follows: (1) How is AI-assisted fall prevention implemented into fall prevention practice, and which factors affect its effective implementation? (2) How is AI-assisted fall prevention aligned with current work practices, how does it influence the work of health care professionals caring for patients at increased risk of falling, and what impact does it have on their work environment? (3) How do patients with increased risk of falling perceive AI-assisted fall prevention, and how does it affect their overall care experience? (4) To what extent does the use of AI-assisted fall prevention reduce fall accidents among patients, and how does this impact clinical and health care–related outcomes? (5) How does the use of AI-assisted fall prevention affect resource usage in hospital wards caring for patients at increased risk of falling?

## Methods

### Overall Design

This project will use a multimethod observational study design [24] integrating qualitative and quantitative approaches to study a real-world implementation of AI-assisted fall prevention across multiple hospital sites. To generate a comprehensive understanding of the implementation and impact of AI-assisted fall prevention in hospital settings, data will be obtained through interviews, observations, surveys, and retrospective data from electronic health records (EHRs).

Research project implementation will take place between 2026 and 2028, as a multicenter study involving up to 5 hospitals within VGR, each encompassing several hospital sites. A longitudinal research design will be used, with data collected at several predefined time points throughout this project’s period. This design facilitates the systematic examination of both implementation processes and their impact over time.

This project encompasses 5 RQs. To support the development of evidence-informed, practice-oriented strategies for AI implementation, 2 learning laboratories will be conducted, providing structured opportunities for stakeholder engagement, knowledge exchange, and cocreation.

An overview of the overall structure, including the individual studies and learning laboratories, is presented in Table 1.

**Table 1.** An overview of this project’s structure.

Study	Research questions	Data material	Data collection; months pre- and post-AI^a^ implementation						Data analyses
			0	3	6	9	12	24	
Study 1: implementation process	How is the AI fall prevention technology implemented into clinical fall prevention practice, and which factors affect its effective implementation?	Interviews (n=55)	N/A^b^	Int^c^	Int	Int	N/A		Qualitative design theory: process evaluation of complex interventions [25] and CFIR^d^ [26]
Study 2: health care professionals’ work; patients’ and relatives’ experiences	How is AI-assisted fall prevention aligned with current work practices, how does it influence the work of health care professionals caring for patients at increased risk of falling, and what impact does it have on their work environment? How do patients with increased risk of falling perceive the AI fall prevention technology, and how does it affect their overall care experience?	Interviews (n=60); observations (200 h); surveys (n=1200)	Sur^e^ 1: baseline	Int; obs^f^	Int; obs	int; obs	Sur 2: follow-up	Sur 3: follow-up	Multiple-method design theory: NPT^g^ [27] and JD-R^h^ model [28]
Study 3: effects on fall rates	To what extent does the use of AI fall prevention technology reduce fall accidents among patients at increased risk of falling, and how does this impact clinical and health care–related outcomes?	Patient health records data (n=1839 at each assessment time point);	Data acq^i^	Data acq	Data acq	Data acq	Data acq	Data acq	Retrospective comparative design
Study 4: resource usage	How does the use of AI fall prevention technology affect resource usage in hospital wards caring for patients at increased risk of falling?	Interviews (n=30)	N/A	Int	Int	Int	N/A	N/A	A system-oriented investment perspective
Learning-laboratory 1‐2	Purpose: to support the cocreation process in the development of implementation strategies.	Health care professionals and patients or patient representatives (n=15)	N/A	N/A	N/A	N/A	Plan ex^j^ 12-month post project start	Plan ex 24-months post project start	N/A

a AI: artificial intelligence.

b N/A: not applicable*.*

c Int: interview.

d CFIR: Consolidated Framework for Implementation Research.

e Sur: survey.

f Obs: observation.

g NPT: Normalization Process Theory.

h JD-R: Job Demands–Resources.

i The table presents a simplified overview of the data collection schedule. Patient health recors data will be collected during 3‑month periods at eight time points: baseline, and 3, 6, 9, 12, 15, 18, and 21 months following implementation of AI‑assisted fall prevention. Full details are provided under the heading ‘Study 3: Effects on Fall Rates.’

j Plan ex: planned execution.

### Theoretical Perspectives

This project will draw on multiple complementary theoretical frameworks. These frameworks provide a comprehensive and multilevel theoretical foundation, enabling analysis of implementation processes, contextual determinants, professional work practices, and organizational as well as patient-related outcomes. For detailed descriptions of the specific theoretical frameworks applied in each study, please refer to studies 1‐4.

### Settings

This project will be conducted in VGR to generate evidence-based knowledge on AI-assisted fall prevention. The research initiative was originally prompted by clinical representatives at Sahlgrenska University Hospital in VGR, who identified the need for rigorous evaluation and validation of the AI technology previously tested and now being implemented across 5 hospital organizations, each comprising multiple hospital sites, covering up to 2400 patient beds. Notably, the implementation of AI-assisted fall prevention is managed entirely by VGR as an operational initiative, independent of and separate from the research activities described in this protocol. This research project does not seek to intervene in or alter the implementation process; rather, it will systematically observe and analyze how the technology is introduced and integrated into routine clinical practice.

### The AI Technology

The AI technology studied in this project is a fall prevention system that uses ceiling-mounted 3D sensors to detect movements in a patient’s room. The sensors emit electromagnetic waves and generate continuous data reflecting motion dynamics in the room. The system does not process video or audio data and does not rely on wearable devices. The movement data are processed by an AI algorithm that classifies predefined movement patterns (eg, bed-exit attempts or standing up from a chair or a wheelchair) into risk-relevant events based on configurable monitoring scenarios and predefined alert criteria. When such an event is detected, the system generates a real-time alert and sends it to health care staff via a mobile app. The system continuously monitors bed and room presence in real time, including in shared rooms. The system is designed to support health care professionals by identifying movement patterns associated with increased fall risk and by providing alerts that enable timely assistance. The system does not initiate clinical actions autonomously; health care professionals receive notifications through the app and decide whether, how, and when to respond to alerts based on the information provided.

The technology is implemented in hospital wards that care for patient groups considered to benefit from this type of fall-prevention measure. Patients are offered the system either upon admission or later during their stay when staff identify a need for additional fall-prevention support. The decision to activate the system for an individual patient is made by health care professionals based on their clinical judgment; staff assess whether the system is appropriate and determine when it should be used.

### Studies

#### Study 1: Implementation Process

This study uses a qualitative design to address RQ1. To explore the implementation process, the Process Evaluation of Complex Interventions framework [25] will be used to study mechanisms of impact and contextual influences, offering a systematic basis for understanding how complex interventions function and how their delivery can be optimized. In parallel, the Consolidated Framework for Implementation Research [26] will guide the identification of multilevel determinants, ranging from intervention characteristics to organizational and individual factors, that influence adoption.

Collected data will include 55 semistructured interviews: 20 with ward managers or division managers and 35 with key persons involved in the implementation (eg, ward-based implementation facilitators and implementation project managers). Data will be collected at 3-, 6-, and 12-month postimplementation (Table 1). Ward and division managers responsible for wards that are planning to implement, or are already implementing, AI-assisted fall prevention are contacted and recruited by the research team. Key persons are then recruited through these ward and division managers, and through designated contact persons and internal communication channels within VGR. Managers and key persons are eligible for inclusion in interviews if they are employed at one of the participating hospitals in VGR, have experience from implementing or working with AI-assisted fall prevention, and have the ability to understand and communicate in Swedish. A purposive sampling will ensure diverse experiences, considering hospital ward specialties. Interviews will be audio-recorded, transcribed verbatim, and analyzed using qualitative methods. The analysis will involve systematic engagement with the transcripts, including careful reading, open coding, and the organization of codes into broader conceptual categories or themes. This process will support the development of a nuanced and empirically grounded understanding needed to answer the RQ.

#### Study 2: Health Care Professionals’ Work and Patients’ and Relatives’ Experiences

This study uses a multiple-method design including qualitative and quantitative methods. In total, this study will include 60 semistructured interviews: 30 with health care professionals and 30 with patients or their relatives. This study will also include microethnographic observations and surveys. To investigate the integration of AI-assisted fall prevention into routine work, the Normalization Process Theory [27] will be applied, focusing on the social and organizational processes that facilitate or hinder the embedding of AI technologies in everyday clinical workflows. Additionally, the Job Demands–Resources model [28] will be used to assess how workplace demands and available resources shape staff well-being, performance, and engagement in the context of AI implementation. Finally, a sociotechnical perspective will be used to analyze how AI reshapes the work practices of health care professionals and influences the experiences of patients and relatives [29].

To address RQ2, this study will include semistructured interviews with 30 nurses and nurse assistants, microethnographic observations [30], and surveys. In total, the observations will include 6‐10 staff members (approximately 2 per ward) and will be conducted over 200 hours, with around 40 hours per ward. Interviews and observations will be conducted at 3, 6, and 9 months post deployment. Surveys will be distributed to all wards planning to implement the AI-assisted fall-prevention technology and that choose to participate in this study, reaching up to 1200 health care professionals across 40 departments at 3 time points: baseline, 12 months, and 24 months post deployment. The goal is to achieve a response rate of 60%, which would correspond to approximately 720 responses per time point. The main outcome investigated in the survey is changes in the work environment over time. The baseline survey will focus on the work environment to establish a clear point of departure before the implementation of AI-assisted fall prevention using COPSOQ III (Copenhagen Psychosocial Questionnaire Version III) [31,32] and Burnout Assessment Tool [33], as well as questions capturing health care professionals’ views on the forthcoming implementation. At follow-ups 1 and 2, the NoMAD (Normalization Process Theory Measure) instrument, based on Normalization Process Theory, is also included [34,35]. The combination of work environment measures and NoMAD enables a deeper understanding of both the organizational impact and staff experiences related to the introduction of the AI technology.

The staff will be recruited via ward and division managers. Throughout this study, purposive sampling will be used to ensure diversity in experiences, with attention to ward specialties across interviews, observations, and surveys. Staff are eligible for inclusion in interviews and observations if they are employed at one of the participating wards where AI-assisted fall prevention technology has been implemented, have experience working with the AI-assisted fall prevention, and have the ability to understand and communicate in Swedish.

To address RQ3, this study will include semistructured interviews with 30 patients and their relatives. Patients will be recruited by staff at each participating ward. If a patient’s ability to provide informed consent is uncertain, no interview will be conducted; instead, a relative may be invited to participate and offer perspectives on their own and the patient’s situation. Patients or family members are eligible for inclusion in interviews if they are either a patient who has been admitted to one of the participating wards where the AI-assisted fall prevention technology has been implemented, or a relative of a patient who fulfills these inclusion criteria but lacks the capacity to provide informed consent. In both cases, the participant must have experience with the AI-assisted fall prevention technology as part of the patient’s care, be able to understand and communicate in Swedish, be 18 years of age or older, and have the capacity to provide informed consent.

Interview transcriptions and field notes will be analyzed using qualitative methods. The analysis will involve systematic engagement with the transcripts, including careful reading, open coding, and the organization of codes into broader conceptual categories or themes. Through this analytic process, a nuanced and empirically grounded understanding will be generated to address the RQ.

Survey data will be analyzed using descriptive and inferential statistics. Continuous variables will be reported as means and SDs or medians with ranges, depending on distribution, while categorical variables will be presented as frequencies and percentages. Internal consistency of the validated scales will be assessed by using Cronbach α. The primary outcome is the change in the work environment over time. To assess changes over time, inferential analyses will be conducted using longitudinal statistical models. Specifically, repeated measurements will be analyzed using linear mixed-effects models (or equivalent methods), which account for within-individual correlations over time. Time will be modeled as a fixed effect, with individuals included as random effects. When the data structure permits, clustering at the ward and/or hospital level will be incorporated as additional random effects to account for hierarchical dependencies. If the sample size is sufficient, subgroup and group-level analyses (eg, ward- and/or hospital-level comparisons) will be performed to explore variation in changes across organizational units. Model assumptions, including normality and homoscedasticity of residuals, will be evaluated using diagnostic plots. The choice of statistical tests and model specifications will be guided by the distribution and scale properties of the outcomes. To assess practical significance, adjusted mean scores of the COPSOQ III scales will be compared with Swedish population benchmarks. A deviation of ±5 points from the reference value will be defined as a minimal important difference, representing the threshold for meaningful change, in accordance with established criteria [32,36]. Missing data will be handled based on the extent and pattern of missingness. Under a missing-at-random assumption, linear mixed-effects models allow inclusion of participants with incomplete repeated measures. If appropriate, multiple imputation will be used as a sensitivity analysis. Participants with insufficient overall response completeness (eg, less than 50% of COPSOQ III items completed) will be excluded from analyses, in line with previous Swedish COPSOQ III studies.

Finally, a mixed methods approach [24] will be used to integrate data generated through qualitative and quantitative methods.

#### Study 3: Effects on Fall Rates

To address RQ4, this study adopts a retrospective comparative design to evaluate differences in fall-related clinical outcomes between patients monitored using AI-assisted fall prevention and those managed with conventional strategies. The main outcome is the fall rate, defined as the number of falls per predefined time period. Data will be extracted from EHRs, including information on fall prevention measures and clinical outcomes. Data collection will encompass all patients admitted to participating wards over 3 months at 8 time points: 0, 3, 6, 9, 12, 15, 18, and 21 months following the implementation of AI-assisted fall prevention. To facilitate pre-post comparisons, control data will also be collected over 3 months from all participating sites, 1 year before the first 4 postimplementation assessments (ie, 0, 3, 6, and 9 months). Data from 12, 15, 18, and 21 months will be analyzed to assess trends in the sustainability of outcomes. A power calculation, based on an estimated fall incidence of 5 falls per 1000 patient days and an average length of stay of 5 days, along with an expected effect size of 0.5, indicates that a sample of 1839 patients per measurement point is required to detect a statistically significant difference (*P*<.05) between the groups. The estimates of fall incidence and average length of stay are based on information from 2024 provided by Sahlgrenska University Hospital, while the expected effect size is derived from previous international research [37]. To achieve a sample of 1839 patients per measurement point within a 3-month timeframe, it is estimated that at least 7 hospital wards need to be included, which is considered feasible within the scope of this project. To ensure diversity across different patient groups, recruitment will involve all hospital wards implementing AI-assisted fall prevention. Data will be analyzed using descriptive statistics to summarize fall prevention measures and fall-related outcomes across study periods and sites (Table 1). The primary analysis will estimate differences in fall incidence between AI-assisted and conventional care using multivariable mixed-effects Poisson regression models, with patient-days included as an offset to estimate incidence rate ratios. Models will adjust for predefined patient- and ward-level covariates, including patient demographics and clinical characteristics, ward type, and other relevant case-mix indicators. To account for the hierarchical data structure, random effects for wards nested within hospitals will be included. Calendar time will be incorporated as a fixed effect to adjust for temporal and seasonal variation across measurement periods. As wards contribute observations at multiple time points, the mixed-effects framework will account for within-ward correlation across repeated measurements. Missing data in covariates will be examined for patterns and extent; when the proportion of missingness exceeds 5%, multiple imputation using chained equations will be applied before model estimation. Although residual confounding cannot be completely excluded in a retrospective design, these analytical procedures aim to reduce bias and strengthen causal interpretation of observed differences.

#### Study 4: Resource Usage

To address RQ5, this study examines how the introduction of AI-assisted fall prevention affects resource usage and cost structures across the health care system. The analysis adopts a system-oriented investment perspective with activity-based costing analysis [38] to allocate and analyze cost structures by resource consumption patterns and cost drivers associated with patient care activities [39]. The analysis will focus on how local investments in fall prevention technology influence resource use and cost distribution across organizational levels and stages of the care pathway [40].

First, potential system effects of the technology will be identified through interviews with stakeholders, such as health care managers (n=10), financial controllers (n=10), and clinical staff (n=10), focusing on how the implementation influences staffing, work processes, and patient flows between different parts of the care system. These insights will be used to map the activities affected by the intervention and to identify relevant cost drivers. Second, the data on affected activities and cost drivers will be used to develop an investment model to analyze how costs and benefits evolve and how they are distributed within the system [41,42]. Finally, the investment model will be used for an accounting-based cost analysis to examine how resource usage changes following the introduction of the technology [43]. Costs will be categorized into direct and indirect costs, as well as fixed and variable costs, in order to analyze how the investment alters the organization’s cost structure and the allocation of resources between technological infrastructure and personnel-related activities. The stakeholder interviews will identify cost drivers and relevant resource use to be included in the investment model. Such quantitative indicators are cost per patient episode, cost per patient day, and distribution of costs across organizational units. The investment model will be used to estimate changes in resource use over time. For the purpose of this analysis, patient health records data collected as part of Study 3 will be analyzed. Data will be collected at 0, 3, 6, 9, 12, 15, 18, and 21 months post implementation and compared with data for care without AI-assisted fall prevention collected predeployment (Table 1).

### Learning Laboratories and AI Implementation Strategies Development

The development of the AI implementation strategies will be an ongoing process throughout this project, informed by this project’s findings. A total of 2 learning laboratories [44] will be held approximately 12 and 24 months after implementation to facilitate knowledge exchange, cocreation, and the development of implementation strategies. The learning laboratories will also provide an opportunity to elicit participants’ perspectives and considerations regarding how to promote patient safety, safeguard good working conditions and professional autonomy, and ensure the appropriate use of resources in the implementation of the AI-assisted fall-prevention system. By creating an environment in which participants’ experiences and viewpoints serve as the starting point, the laboratories will support a deeper understanding of the technology’s role in practice. Each laboratory will involve around 15 participants, including nursing staff, patient representatives, and patients or family members with experience in the AI technology. Participants will be recruited through reference groups, VGR’s internal channels, and patient organizations. The objective is to (1) identify the need for implementation strategies, (2) evaluate the applicability of this project’s results, and (3) cocreate implementation strategies by integrating research insights with practical experience. Learning laboratories will be documented through notes and audio recordings, which will be analyzed using qualitative methods.

### Ethical Considerations

This research involves the processing of sensitive personal data related to patients’ health and has been reviewed and approved by the Swedish Ethical Review Authority in accordance with the Act (2003:460) on Ethical Review of Research Involving Humans (ref. 2025‑03701‑01 and 2025‑08850‑02). This project will adhere to the ethical principles in the Declaration of Helsinki and national ethical guidelines, ensuring information, consent, confidentiality, and participant safety. In addition, this study draws on ethical principles for the use of AI in health care, including sustainability, human-centeredness, inclusiveness, fairness, and transparency [45], which provide an approach for reflecting on and interpreting the use of AI in the clinical context. Patient representatives participating in the learning laboratories are compensated for an average of 10 hours of involvement, corresponding to approximately US $165.

All participants will receive both oral and written information before participation. They will be informed that participation is voluntary and can be withdrawn at any time without affecting their work or care. Informed consent will be obtained, and all data will be handled to ensure no unauthorized access. Identifiable information will be pseudonymized, and personal data will be processed in compliance with the General Data Protection Regulation (2016/679), in consultation with the data protection officer at Halmstad University.

The extraction and linkage of EHR data are conducted under the approved ethical permits and in accordance with the regional health care authority’s data governance procedures and General Data Protection Regulation. Patients’ personal identity numbers will be used solely to enable linkage of retrospective medical record data. After the linkage is completed, each patient will be assigned a randomly generated numerical code. All processing of data containing personal identity numbers will occur within the secure firewalls of the VGR’s regional data warehouse at the respective hospital administration. The linkage will be performed by the administration’s IT department, which has substantial experience in managing sensitive personal data. The researchers will only receive data that contains no personal identifiers.

All collected personal data, as well as raw data, are stored in Sunet Drive (The Swedish Research Council), separately from the pseudonymized data. Sunet Drive is considered a secure environment for managing personal data, and the University uses its own local instance of the platform. Data are managed and archived in accordance with the applicable regulations governing the handling and archiving of research records at Halmstad University.

The primary ethical consideration in this project relates to the inclusion of patients with cognitive impairment. While such inclusion presents challenges related to informed consent and safeguarding, the potential benefits are judged to outweigh the risks. Patients are recruited by staff at each participating ward. Contact nurses recruit patients during the hospital stay and ensure that informed consent is obtained. The patient’s capacity to provide consent is assessed when needed in consultation with health care staff and a relative. If there is uncertainty about a patient’s ability to consent, the researchers will not conduct the interview. In such cases, a relative may instead be invited to participate to provide perspectives on the patient’s situation. Participation is entirely voluntary, and patients are informed that their decision to participate or decline will not affect their current or future care, treatment, or relationship with health care staff. They are also informed that they may withdraw from this study at any time without providing a reason and without any consequences for their care. Patients or relatives who wish to participate are contacted by the researchers after the hospital stay or once the patient is medically stable. At that time, the researcher again provides written and verbal information about this study and obtains documented consent, either digitally or on paper. If a patient has shown interest in participating but their capacity to consent remains uncertain, the researcher will refrain from conducting the interview. Respect for autonomy will be ensured by adapting the consent process to each participant’s cognitive abilities and providing support to aid understanding. Patient well-being will take priority over data collection, and participation will remain strictly voluntary. Researchers will remain sensitive to circumstances where interviews and observations may be inappropriate. If a patient is assessed as lacking the capacity to provide informed consent, the patient will not be included in interviews or observations.

Discussions about patient safety in interviews may give rise to sensitive issues, such as feelings of blame or inadequacy among health care professionals. Ethical dilemmas may also emerge when staff, following AI-generated recommendations, are required to prioritize 1 patient over another in situations where the risks appear similar, something that may be experienced as morally challenging. To safeguard staff participation within the workplace setting, several measures will be implemented to ensure that participation in this study does not lead to perceived threats to professional integrity, especially in matters relating to the prioritization of patient safety interventions. Participation will be entirely voluntary and based on written informed consent. Recruitment procedures will be designed to minimize any perceived pressure from managers or colleagues. Line managers will not be informed about who elects to participate or declines participation. Participation or nonparticipation will have no bearing on employment conditions, performance appraisal, career progression, or professional relationships. All data will be handled in strict confidence, pseudonymized at the point of transcription and statistical analysis, and reported at an aggregated level to prevent identification of individual staff members. Interviews will be conducted in a private setting. The survey will be distributed to participants’ work email addresses and is intended to be completed during working hours. Participation remains entirely voluntary, and managers will not have access to information about who has responded or declined. These procedures are intended to mitigate any potential risks associated with participation.

Observations on the wards will be carried out with full respect for the integrity and privacy of both patients and health care professionals. Informed consent will consistently be obtained from health care staff, patients, and their family members before any observations take place. Obtaining informed consent in clinical environments can be challenging, and researchers must remain attentive to the circumstances present in each situation. If there is any uncertainty regarding a patient’s capacity to provide consent, the observation will not be conducted.

Another challenge associated with observational work is the risk that participants may develop expectations of the researcher that are not compatible with the research role, particularly during more informal interactions over time. To minimize this risk, observations will be carried out over a limited period, and the risk is assessed as low.

## Results

### Overview

The results are expected to have the potential to inform large-scale initiatives in other regions, where similar AI technologies are under consideration. Therefore, contact has been initiated with other Swedish regions, which are in the early stages of implementing similar AI technology. Establishing a national network is expected to strengthen the scientific foundations of AI-driven fall prevention and improve health care quality. Thus, we will also establish collaboration with similar national and international projects on AI-assisted fall prevention.

### Project Organization

A project group and a steering group have been established to facilitate the operationalization of this project. This project’s group members consist of researchers from Halmstad University, University of Gothenburg, and the University of Skövde, who will be responsible for executing this project’s design. The researchers from Halmstad University are organizationally situated within the Healthcare Improvement Research Group at Halmstad University, whose work aims to validate various AI uses in health care and provide evidence-based knowledge [16]. Over the years, the group has built strong expertise around the development, evaluation, and implementation of AI-supported health innovations in various health care contexts. The research has identified key knowledge gaps [46-49] and contributed to both theoretical insights and frameworks for implementation [14,50-52]. It has also advanced understanding in real-world cases of implementation and the integration of AI in health care [15,17,53,54]. This body of work has significantly contributed to a deeper understanding of the challenges and key factors influencing the adoption and long-term sustainability of AI in health care.

To ensure a strong practical integration, this project is guided by an interdisciplinary steering group consisting of researchers from Halmstad University and both researchers and clinicians from VGR, who together encompass expertise from various scientific fields and different study designs and methods. The steering group members from VGR will ensure that the research aligns with the needs of the partner organization and facilitates both accessibility to the health care setting and the dissemination of results. Through this collaboration with VGR and Halmstad University, this project ensures that the findings are applicable and scalable within Swedish health care systems.

In addition, this project includes two reference groups: (1) a group of health care professionals who are affected by the introduction of AI-assisted fall prevention, and (2) a group of patient representatives, patients, and their relatives who either experienced AI-assisted fall prevention or are affiliated with patient organizations for whom falls are a central concern. The reference groups aim to ensure that the research remains relevant from both a health care professional and a patient perspective. These groups, which will be recruited at the beginning of this project, will actively contribute both to the interpretation of this study’s results and to the development of strategies for future implementation. Participants in this project’s 2 learning laboratories will be recruited from these groups. Insights from the reference groups will ensure that the implementation strategies are based on real-life experiences, considering both the practical challenges faced by health care professionals and the needs of patients.

### Project Implementation

This project is scheduled to start on January 1, 2026, and end on December 31, 2028. Before the official start, a prestudy phase was initiated and conducted during 2025.

Throughout this project management, the focus will be on maintaining structured communication, facilitating the work of reference groups and collaborations, and implementing a comprehensive dissemination and monitoring strategy to maximize this project’s scientific and practical impact.

The dissemination of research will take place throughout this project’s duration and will target six groups: (1) health care staff in VGR, (2) health care staff nationwide, (3) patients and their relatives, (4) the research community, (5) citizens and residents, and (6) employers and trade unions.

## Discussion

### Significance and Scientific Novelty

This research project responds to the critical need for robust, evidence-based knowledge on the implementation and effects of AI-assisted fall prevention in hospital environments. While AI technologies are increasingly used as tools to enhance patient outcomes, there is a scarcity of rigorous studies examining their real-world performance, particularly in complex clinical environments [47]. By situating this research within one of Europe’s largest health care regions and embedding it in the full-scale implementation of AI-assisted fall prevention across 5 diverse hospital settings, this project offers a rare opportunity to generate contextually grounded and broadly relevant knowledge. The setting enables the systematic investigation of implementation processes, clinical outcomes, and the interplay among technology, health care professionals, and patients. The use of a multimethod observational research design [24] integrating qualitative and quantitative approaches enhances the generalizability and scalability of the findings.

The current project is expected to generate a level of evidence on AI-assisted fall prevention that goes beyond what is currently available from the limited number of previous research [11-13] and pilot studies [20-22]. In particular, the current project will contribute new knowledge into its impact on patient safety, the work of health care professionals, patient experiences, and resource usage, as well as the conditions necessary for effective implementation.

A central strength of this project lies in its collaborative approach, engaging health care staff and patients in the codevelopment of practical strategies to support hospitals in implementing AI-assisted fall prevention. This collaborative approach will help ensure that research findings are not only scientifically rigorous but also directly translatable into sustainable clinical practice. This is particularly important given the growing integration of AI in health care, where the rapid pace of technological advancement often outpaces the availability of rigorous, real-world evidence [55]. By bridging this gap, the current project will contribute to the adoption of AI in health care guided by high-quality evidence, which both promotes patient safety and is responsive to the needs of patients, relatives, and health care professionals.

The results of this project will be relevant to a wide range of stakeholders, including patients, health care professionals, employers, trade unions, researchers, and policymakers. Conducted in collaboration with VGR, one of Sweden’s largest health care providers, serving approximately 1.8 million residents and employing approximately 50,000 health care professionals, this study builds on a large-scale, real-world implementation of AI-assisted fall prevention across a complex health care system. This context provides a rare opportunity to investigate the intervention’s impact at scale and to generate findings that are directly applicable and scalable across Swedish health care settings. Although rooted in the Swedish context, this study’s insights will be relevant internationally, particularly for health care systems considering the introduction or expansion of AI-assisted fall prevention.

### Strengths and Limitations

The primary strength of this project lies in the close collaboration between Halmstad University, University of Gothenburg, University of Skövde, and hospitals in VGR. This partnership, grounded in clinical relevance and clear health care needs, facilitates access to real-world settings and strengthens the significance of the research by directly addressing the urgent demand for evidence on the implementation and effects of AI-assisted fall prevention [56].

An additional strength of this project is its multicenter, multimethod, and robust observational design, which supports longitudinal studies by using diverse data collection methods, such as quantitative measurements, interviews, and observational data, and gathering large volumes of data over time. This design enables a comprehensive investigation of changes in both the implementation process and the impact of AI-assisted fall prevention throughout this study’s period. The combination of qualitative and quantitative approaches allows not only for the precise measurement of effects but also for a deeper understanding and explanation of the underlying mechanisms driving these effects. Such longitudinal multiple methods designs are particularly valuable for capturing dynamic processes in real-world health care settings, although they are also resource-intensive and logistically demanding [24,57]. Furthermore, the current project benefits from extensive data capturing a wide range of patient health conditions across diverse care settings in this multicenter context, including both large university hospitals with complex care environments and smaller regional hospitals with different organizational structures, thereby enhancing the breadth and depth of the analysis [55].

This project’s large scale and complexity present inherent challenges, including the risk of an extended study timeline. The research is contingent upon the implementation of AI-assisted fall prevention by the hospitals within the VGR, indicating that the project team operates to some extent under external dependencies. Without successful and timely deployment by the regional hospitals, the research cannot proceed as planned. This dependency introduces uncertainty regarding the pace and consistency of implementation across participating in this multicenter study. It is therefore important to clarify that the research is conducted in parallel with an implementation that has already been decided by the health care organization; this study does not direct, influence, or steer this implementation in any way, and data collection can only begin once the hospitals themselves have initiated the process. This project does not seek to evaluate the hospital’s implementation efforts or the technical product, but to examine scientifically how the technology affects everyday clinical practice. The findings will thus provide insights into the effects of introducing AI technology in a specific health care context, rather than serving as a product evaluation or an assessment of the implementation process.

While the use of diverse methods, both qualitative and quantitative, enables a comprehensive exploration of multiple perspectives and dimensions of the implementation and impact, it also necessitates a broad range of methodological competencies, which this project’s team possesses. However, managing large volumes of data across varied methods is resource-intensive and poses logistical challenges. Moreover, there is some uncertainty regarding the exact profile of participating health care settings; the sample may end up being relatively homogeneous or, alternatively, highly heterogeneous, which may affect the generalizability of findings. These challenges may be amplified in a multicenter study, as local organizational structures and routines can influence implementation and follow-up differently across sites [55].

Another limitation concerns the inclusion of informants with cognitive impairments and patients who may have transient interactions with the health care system. Recruiting these participants presents ethical and practical challenges, potentially impacting data completeness. These factors require careful consideration in study design and analysis to ensure valid and reliable conclusions [57].

This project aims to develop implementation strategies for AI-assisted fall prevention. A limitation is that the development of these strategies is planned before the effects of the intervention are known, which risks a solution-driven rather than needs-driven approach. By first examining the experiences of patients, relatives, and health care professionals, we aim to ensure that the resulting strategies are grounded in real-world practice and actual needs. Our goal is to support hospitals, when they adopt such technologies, in ways that safeguard patient safety, support good working conditions, and ensure appropriate use of resources. As AI-assisted fall prevention is an emerging trend in Sweden and internationally, empirically informed strategies from this project may guide other regions undertaking similar implementation processes, regardless of the specific AI product.

Given the complexities involved in real-world integration of AI-assisted fall prevention, there will be contextual factors, unreported user experiences, or emergent organizational dynamics that cannot be measured. Despite the inherent limitations of this study’s design, it offers multiple methods that can capture and triangulate findings to help reveal nuanced patterns across the wide ranged data sources and how they can be understood.

## Abbreviations

AI: artificial intelligence
COPSOQ III: Copenhagen Psychosocial Questionnaire Version III
EHR: electronic health record
NoMAD: Normalization Process Theory Measure
RQ: research question
SAFE: Safe AI-Assisted Fall Prevention Through Evidence
VGR: Västra Götaland Region
